# Denoising Respiratory Sinus Arrhythmia of Pulse-to-Pulse Interval Signals Extracted from Photoplethysmogram with an Autoregressive Moving Average Model

**DOI:** 10.3390/s26103048

**Published:** 2026-05-12

**Authors:** Shing-Hong Liu, Chien-Kai Lin, Xin Zhu, Jia-Jung Wang, Yu-Lun Hsu, Kuo-Li Pan

**Affiliations:** 1 Department of Computer Science and Information Engineering, Chaoyang University of Technology, Taichung City 41349, Taiwan; shliu@cyut.edu.tw (S.-H.L.); s11427603@gm.cyut.edu.tw (C.-K.L.); 2 Department of AI Technology Development, M&D Data Science Center, Institute of Integrated Research, Institute of Science Tokyo, Tokyo 101-0062, Japan; zhu.xin@tmd.ac.jp; 3 Department of Biomedical Engineering, I-Shou University, Kaohsiung City 84001, Taiwan; 4 Bachelor’s Program of Sports and Health Promotion, Fo Guang University, Jiaoxi 26247, Yilan County, Taiwan; ylhsu@gm.fgu.edu.tw; 5 Division of Cardiology, Department of Internal Medicine, Chang Gung Memorial Hospital, Chiayi Branch, Chiayi City 613, Taiwan; pankuoli64@gmail.com; 6 College of Medicine, Chang Gung University, Taoyuan City 333, Taiwan; 7 Heart Failure Center, Chang Gung Memorial Hospital, Chiayi Branch, Chiayi City 613, Taiwan

**Keywords:** pulse-to-pulse interval, respiratory sinus arrhythmias, autoregressive moving average model

## Abstract

**Highlights:**

**What are the main findings?**

- Evaluating the performance of individual subject models and a general model.
- Selecting appropriate model orders for different individual subject models and the general model.
- Evaluating whether different control breathing rates (CBRs) generated the respiratory sinus arrhythmia (RSA) energies coupled in the PPI signals.
- Evaluating whether the proposed individual subject ARMA model and the general ARMA model significantly attenuate RSA energy in the frequency-domain parameters of PRV analysis.

**What are the implications of the main findings?**

- The performance of individual subject models does not significantly differ from that of the general model for removing the RSA energy coupled in the raw PPI signals.
- The general model could be embedded in an edge computing system in the future.

**Abstract:**

Background: Pulse rate variability (PRV), a critical biomarker of autonomic nervous system (ANS) function, is typically evaluated using the pulse-to-pulse interval (PPI) signal extracted from a photoplethysmogram (PPG). Although PPGs have been widely used in wearable devices, the PPI signal is easily affected by motion artifacts or respiratory sinus arrhythmias (RSAs). These disturbances affect the accuracy of PRV for evaluating ANS function. The aim of this study was to remove the respiratory signals from raw PPI signals with an autoregressive moving average (ARMA) model. Methods: An R-wave to R-wave interval (RRI) sequence was extracted from the electrocardiogram (ECG). A self-made measurement system was used to record PPG, ECG, and respiratory signals. Nineteen healthy adults were recruited and requested to breathe with a spontaneous breathing rate (SBR) and control breathing rates (CBRs) (6, 18, and 30 breathing rate per minute, BRPM). Their ECG, PPG, and breathing signals were recorded for 6 min under different CBRs. The measurement was performed twice, i.e., eight measurements were performed. The raw RRI(t) and PPI(t) signals of 4 Hz were segmented into samples of one minute and shifted by 30 s. Thus, a subject had 80 samples, and there were 10 samples for each BRPM. RSA-free RRI signals were generated by a spectral method to filter RSA from raw RRI(t) to produce the target RRI(t). We proposed the individual subject ARMA models trained by samples with the maximum mean absolute errors between the target RRI(t) and raw PPI(t) (MAE_RAW_s) of each subject, and the general model trained by samples with all maximum MAE_RAW_s of all 19 subjects. Results: The mean absolute errors between the target RRI(t) and $\tilde{PPI}(t)$ predicted by the individual subject ARMA models (MAE_Subject-Models_) and general ARMA model (MAE_General-Model_) were used to evaluate the performance of the two models. The results for the MAE_Subject-Models_ and MAE_General-Model_ were 132.5 ± 59.1 ms and 137.8 ± 67.8 ms, respectively, with no significant difference. MAE_Subject-Models_ and MAE_General-Model_ were compared with MAE_RAW_s, whose attenuations (ATTs) were 28.5 ± 13.1% and 27.8 ± 12.6%, respectively. Conclusions: The two proposed models are capable of removing the RSA energy coupled in the raw PPI signals.

## 1. Introduction

Photoplethysmography (PPG) has gained considerable attention as a practical alternative for heart rate (HR) monitoring, particularly in wearable applications. The heart rate variability (HRV) is a crucial indicator of autonomic nervous system (ANS) function, reflecting the dynamic interplay between sympathetic and parasympathetic modulation regarding cardiac activity [1]. It refers to the physiological phenomenon of variation in the time interval between consecutive heartbeats, approximately equivalent to the R-wave to R-wave interval (RRI) in an electrocardiogram (ECG). Sympathetic nerve activity increases HR, while parasympathetic nerve activity slows HR, and the withdrawal of parasympathetic activity leads to an increase in HR.

The sympathetic nerves intensify cardiac activity by increasing the HR and decreasing HRV. Contrary to sympathetic nerves, the parasympathetic nerves decrease the HR and increase HRV. The ANS responses are commonly assessed through time- and frequency-domain parameters of HRV [2]. HRV has become a valuable biomarker in clinical and research settings for evaluating stress, fitness, and disease prognosis [3]. While HRV indices are widely used as noninvasive markers of ANS activity, they do not provide a direct or exclusive measure of sympathetic and parasympathetic balance. Frequency-domain components such as LF and HF are influenced not only by autonomic modulation but also by respiration, baroreflex mechanisms, and other physiological factors, which can confound their interpretation. Moreover, the assumption that LF reflects sympathetic activity and HF reflects parasympathetic activity has been debated, limiting the reliability of HRV indices as precise indicators of ANS function [4,5].

PPG measures peripheral blood-volume changes using optical sensors and is widely integrated into wristbands, rings, and smartwatches [6,7,8]. The pulse-to-pulse interval (PPI) is extracted from PPG signals, and its sequence is very close to its RRI counterpart. Thus, pulse rate variability (PRV) has been replaced with HRV to describe ANS functions [9,10]. However, PPG waveforms are easily affected by vascular compliance, peripheral vascular resistance, sensor displacement, skin tone, ambient light variations [11,12], and motion artifacts [13]. Peralta et al. evaluated the precision of PRV with HRV derived from ECG as a reference. They evaluated the five different points of the PPG waveform and found that the middle-amplitude point, apex point of the first differentiation, and tangent intersection point were the most suitable fiducial points for the HR monitor, resulting in the lowest relative errors between the PR and HR indices [14]. However, the respiratory sinus arrhythmia (RSA) is modulated in the PPI or RRI sequences. This may affect the accuracy of PRV or HRV parameters. Moreover, this noise usually cannot be removed by infinite impulse response (IIR) or finite impulse response (FIR) filters. To address these challenges, traditional signal processing techniques have been applied to RRI signals, such as the adaptive filter method [15,16], principal component analysis (PCA) [17], and empirical model decomposition (EMD) [18,19]. For the adaptive filter and PCA methods, the least mean squares (LMS) loss function was used to train the model. In these approaches, the synchronous respiratory signal served as the reference and had to be measured simultaneously, which is a major limitation of both methods. EMD was used to decompose RRI signals, where the respiratory component is expected to appear in one of the intrinsic mode functions (IMFs). Therefore, the EMD method also requires a synchronous respiratory signal as the reference. Moreover, because the RRI signal is non-stationary, this method requires additional post-selection of components to identify which IMF contains the largest respiratory energy. Based on the above discussion of previous studies, developing a method to remove RSA energy coupled in the RRI signal without requiring a synchronous respiratory signal or post-selection of components remains a challenge for realizing PRV applications in real-world settings. State-space models have been applied for modeling or denoising the dynamic physiological signals, such as for forecasting sleep apnea by HRV [20,21] and denoising neural data [22].

PRV or HRV measurement involves several parameters categorized into time- and frequency-domain analyses, each providing unique insights into autonomic nervous system function. Time-domain parameters analyze variations in successive PPI or RRI signals, with common metrics including the standard deviation of NN intervals (SDNN) and the root mean square of successive differences (RMSSD) [1,2]. Frequency-domain analysis decomposes PPI or RRI signals into different frequency bands, such as very low frequency (VLF, 0.003–0.04 Hz), low frequency (LF, 0.04–0.15 Hz), and high frequency (HF, 0.15–0.40 Hz), which reflect sympathetic and parasympathetic activity, respectively [2]. Heart rate (HR) is influenced by various physiological, psychological, and environmental factors, all of which contribute to its dynamic regulation. Respiration and HR are closely linked through a phenomenon known as respiratory sinus arrhythmia (RSA), in which HR increases during inhalation and decreases during exhalation [21]. Thus, Tan et al. used RRI signals to forecast whether the sleep apnea is happened [23]. This synchronization is primarily mediated by the autonomic nervous system, which adjusts HR in response to respiratory cycles to optimize gas exchange and circulatory efficiency. The RSA energy is influenced by factors such as breathing rate and tidal volume; for example, slower and deeper breathing tends to enhance RSA, whereas rapid breathing may attenuate it.

During conditions such as exercise or obstructive sleep apnea, respiratory rate changes are accompanied by corresponding variations in RRI. Both the RRI sequence and the R-wave amplitude sequence have been used to predict sleep apnea [22]. Consequently, HRV analysis is typically limited to resting RRI signals. Many wearable devices, such as the Apple Watch and Galaxy Watch, measure the PRV function under a spontaneous breathing rate (SBR) at resting state. However, after exercise, users typically sit down and perform controlled breathing (CBR) to quickly recover. During this period, RSA energy becomes coupled with the PPI signal, which may reduce the accuracy of PRV analysis. Therefore, developing methods to remove respiratory influences from PPI or RRI signals remains an important research direction. To address the impact of RSA energy on PPI and RRI signals, this study employed an autoregressive moving average (ARMA) time-series model to remove the RSA energy coupled in the raw PPI signal, thereby predicting the intrinsic PPI signal.

However, ARMA models face significant limitations, primarily centered on the requirement for stationary, linear data, and challenges in model identification. Common issues include sensitivity to outliers, poor performance with nonlinear or non-stationary data, error accumulation in long-term forecasting, and the difficulty of selecting appropriate orders. In this study, we proposed the individual subject ARMA models (ARMA_individual_model_s) and the general ARMA model (ARMA_general_model_). To support model development and evaluation, a self-made multi-channel measurement system was used to synchronously measure ECG and PPG and respiratory signals under controlled and normal breathing. Data were collected from 19 participants to ensure subject-level variability. The key contributions of this study are as follows: (1) The proposed method does not require synchronous respiratory signals. (2) The CRB energy coupled in raw RRI signals was filtered by the spectral method. The filtered RRI signals were used as the target output of ARMA models. (3) Both ARMA_individual_model_s and ARMA_general_model_ were developed and their performances were compared. (3) Appropriate model orders were selected for both ARMA models. (4) The performance of the proposed models was evaluated under different CBRs. (5) The accuracy of three frequency-domain parameters extracted from the predicted PPI signals was evaluated.

## 2. Materials and Methods

**Figure 1:**
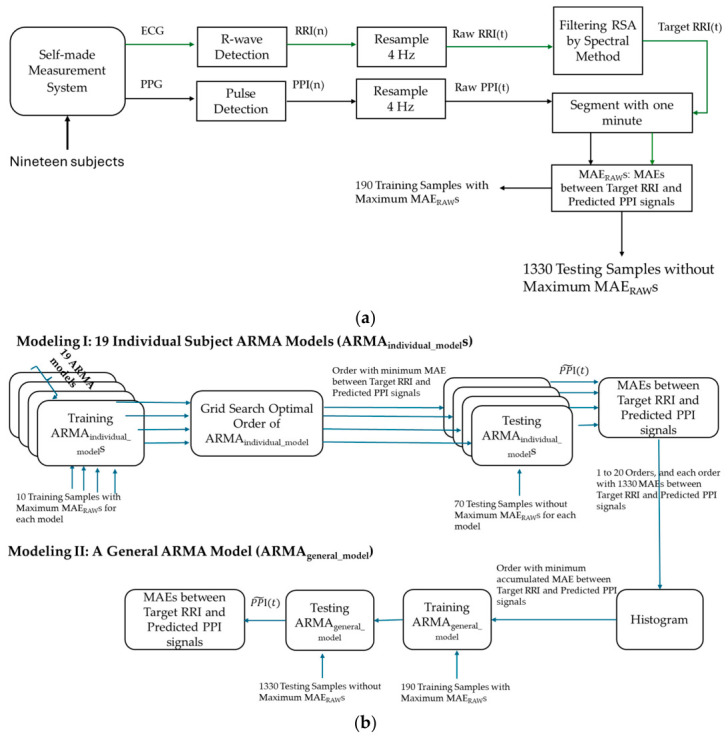
(**a**) The flowchart for generating the training and testing samples from the ECG and PPG signals. (**b**) The flowchart for training and testing the two models, the ARMA_individual_model_s and ARMA_general_model_.

Figure 1 depicts the flowchart of this study for removing the RSA energy coupled in the raw PPI signals using the 19 ARMA_individual_model_s and ARMA_general_model_. Figure 1a shows the flowchart for generating the training and testing samples from the ECG and PPG signals. Nineteen subjects were recruited in this study. A self-made measurement system was used to measure the ECG, PPG, and respiratory signals. Then, the R-waves of the ECG and main peaks of PPG were detected to generate the raw RRI(n) and PPI(n) sequences, which were coupled with respiratory information. Both synchronous sequences were affected by the same RSA and were subsequently resampled at 4 Hz to generate the raw RRI(t) and PPI(t) signals. A spectral method was then applied to remove the respiratory component coupled in the raw RRI(t) signals. The resulting filtered RRI(t) signals and the raw PPI(t) signals were used as the target output and input, respectively. Both signals were segmented into samples of one minute and shifted by 30 s. The subjects were seated in a resting state under spontaneous breathing (SBR) and three controlled breathing rates (CBRs), with each condition lasting 6 min. Physiological signals were recorded throughout each phase. The experiment was repeated twice with a one-week interval, resulting in eight measurements per subject. Each measurement comprised 10 samples collected under different breathing conditions. Subsequently, the mean absolute error (MAE_RAW_) between the target RRI(t) and the original PPI(t) was computed for all samples. For each subject, the measurement comprising 10 samples with the highest average MAE_RAW_ was selected for training the individual subject ARMA model, whereas the remaining 70 samples were used for testing. For the general ARMA model, 190 samples from nineteen measurements were used for training, while the remaining 1330 samples were used for testing. Thus, the resulting training-to-testing ratio was 1:7.

Figure 1b shows the flowchart for training and testing the two models, the ARMA_individual_model_ and ARMA_general_model_. For the ARMA_individual_model_, each model was first trained using the 10 samples with the maximum MAE_RAW_ for that subject, with model orders ranging from 1 to 20. The model was then tested using the remaining 70 samples. The optimal order of each model was determined using a grid search over this range. The order that yielded the minimum MAE between the target RRI(t) and the predicted $\tilde{PPI}(t)$, denoted as the minimum MAE_Subject-Model_, was selected as the order of the ARMA_individual_model_. The testing MAE_Subject_Model_s under the optimal order were compared with the ARMA_general_model_.

For the ARMA_general_model_, all the MAE_Subject_Model_s of the same order were accumulated. A histogram was used to determine the order of the ARMA_general_model_. If an order yielded the smallest accumulated MAE_Subject-Model_, it was selected as the order of the ARMA_general_model_. Subsequently, the samples with the maximum MAE_RAW_ for each subject (totaling 190 samples) were used to train the ARMA_general_model_, while the remaining 1330 samples were used for testing.

### 2.1. Experiment Protocol

**Figure 2:**
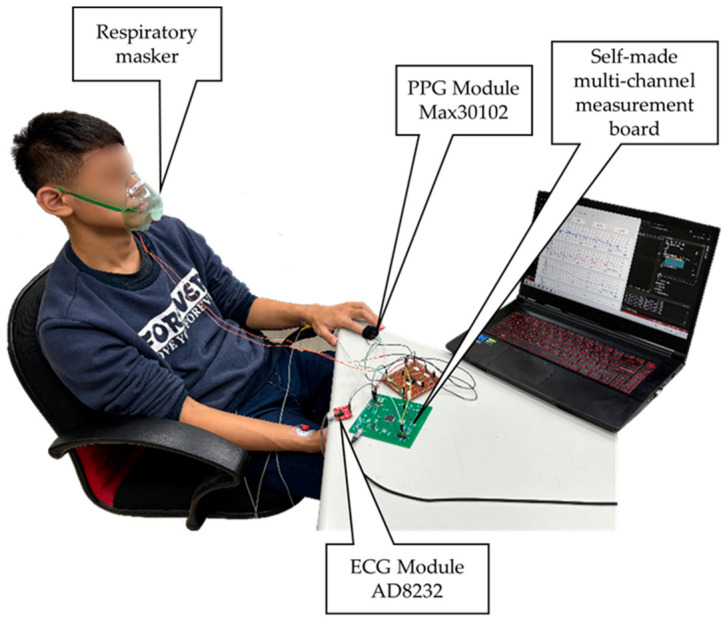
A real photo of the experiment.

Nineteen subjects (11 males and 8 females) were recruited for this study. Their ages, weights, and heights were 20.9 ± 1.4 years (19–24 years), 60.2 ± 13.8 kg (43–89 kg), and 164.3 ± 7.9 cm (150–178 cm), respectively. This experiment was approved by the Institutional Review Board (IRB) of the E-DA Hospital, Kaohsiung, Taiwan (No. EMRP-111-013). The IRB is organized and operates in accordance with Good Clinical Practice and the applicable laws and regulations. Figure 2 shows a real photo of the experiment. The experiment procedure is described as follows:

- The subjects were attached to three electrodes for ECG measurement: the red electrode (negative input) was placed on the right wrist, the yellow electrode (positive input) on the left wrist, and the green electrode (ground) below the right rib.
- A MAX30102 sensor was placed on the tip of the left index finger to measure the PPG.
- The subjects wore an oxygen mask, and the respiratory signals were recorded.
- The subjects sat down on a chair at resting state at an SBR for 6 min. Then, subjects were requested to breathe at CBRs, including six breathing rate per minute (6 BRPM), eighteen breathing rate per minute (18 BRPM), and thirty breathing rate per minute (30 BRPM). In each phase, signals were recorded for 6 min.
- After completing the above procedures, the entire experiment was repeated twice with a one-week interval. Thus, each subject performed 8 measurements.

### 2.2. Self-Made Measurement System

**Figure 3:**
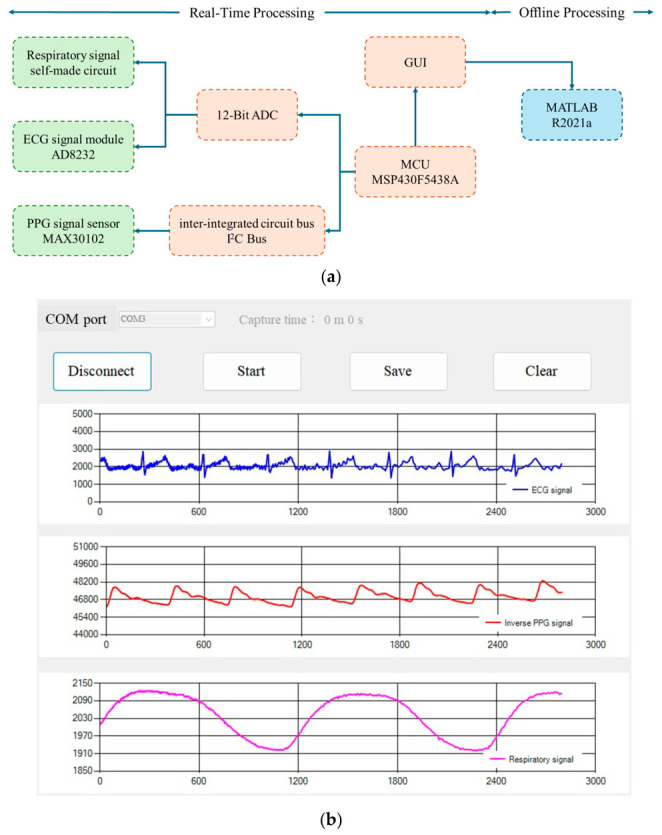
(**a**) The diagram of the self-made measurement system, including a self-made circuit for respiratory measurement, an ECG module (AD8232), and a PPG module (MAX30102). The MCU is MSP430F5438A. The sampling rate is 500 Hz. (**b**) The GUI of the self-made measurement system. The upper row illustrates the ECG, the middle row the PPG, and the lower row the respiratory signal.

Figure 3a illustrates the self-made measurement system. A Zener diode (DO-35, Comchip Technology Co., Ltd., Yingge District of New Taipei City, Tai-wan) was used to detect the respiratory signal, with a gain of 101, and the bandwidth was 0.213–10.26 Hz. The ECG was recorded by an AD8232 module (Analog Device, Wilmington city, MA 01887, USA), and the PPG was measured using a MAX30102 module (Maxim Integrated TM, San Jose, CA, USA). The bandwidth of AD8232 was set between 7 and 24 Hz. The gain was 1100. The red LED of MAX30102 was used to detect the PPG, with a current of 51 mA and a resolution of 18 bits. The ECG and respiratory signals were digitized using a 12-bit Analog-to-Digital Converter (ADC) for further processing. The microcontroller unit (MCU) of the multi-channel measurement board [24] was a 16-bit microcontroller (MSP430F5438A, Texas Instruments TM, Dallas, TX, USA), which handles data management and communication through an inter-integrated circuit (I2C) bus with a MAX30102 module. The MCU interfaces with a graphical user interface (GUI) for real-time visualization, as shown in Figure 3b; the upper row illustrates the ECG, the middle row the PPG, and the lower row the respiratory signal. The data was further analyzed using MATLAB R2021a (MathWorks Corp., USA). The sampling rate of this system was 500 Hz.

### 2.3. Digital Signal Processing

This study employed a third-order Butterworth band-pass filter to preprocess ECG signals. The passband frequency was 5–50 Hz. This bandwidth could enhance the R waves. A third-order Butterworth band-pass filter was used to preprocess the PPG signals. The passband frequency was 0.4–5 Hz. This bandwidth could smooth the dicrotic notch of the PPG. The R waves of the ECG were detected using the Pan–Tompkins method [25]. Then, the maximum slope of the PPG waveform was detected from the differential PPG [14]. The RRI was calculated as the temporal difference between consecutive R waves, as shown in Equation (1).

(1) $${RRI}_{i}=t_{R(i+1)}-t_{R(i)},$$

where *t_R_*_(*i+*1)_ and t*_R_*_(*i*)_ are the times of *i +* 1*^th^* and *i^th^* R waves. If RRI*_i_* is not in [0.5 s, 1.0 s], the RRI*_i_* is considered an abnormal beat and replaced with 0.5*(RRI*_i_*_−1_ + RRI*_i_*_+1_). In breathing-controlled experiments, respiratory components were controlled to 6, 18, and 30 BRPM (approximately 0.1 Hz, 0.3 Hz, and 0.5 Hz), respectively.

A single fiducial point per pulse was used as the maximum-slope detection method. For each pulse cycle bounded by two adjacent troughs $\left(n_{F_{i}},n_{F_{i+1}}\right)$, the fiducial point $n_{A_{i}}^{*}$ was defined as the time index on the rising edge where the first derivative of the signal reached its maximum value, as expressed in Equation (2). This approach identifies the point of maximum upward slope corresponding to the onset of the systolic upstroke, providing high temporal precision and robustness against the amplitude variation of PPI sequences.

(2) $$n_{A_{i}}^{*}=\arg{max}_{\;n\in [n_{F_{i}},n_{F_{i+1}}]}\left\{\frac{{dx}_{PPG}(n)}{dn}\right\}.$$

The raw PPI was obtained as the temporal difference between two consecutive fiducial points as shown in Equation (3).

(3) $${PPI}_{i}=t_{P(i+1)}-t_{P(i)},$$

where *t_P_*_(*i+*1)_ and *t_P_*_(*i*)_ are the times of the (*i+*1)*^th^* and *i^th^* fiducial points. If PPI*_i_* is not in [0.5 s, 1.0 s], the PPI*_i_* is considered an abnormal beat and placed with 0.5*(PPI*_i_*_−1_+PPI*_i_*_+1_). The raw PPI sequence was synchronized with the raw RRI sequence for comparative analysis and subsequent model training.

### 2.4. Raw and Target RRI and Raw PPI Signals

**Figure 4:**
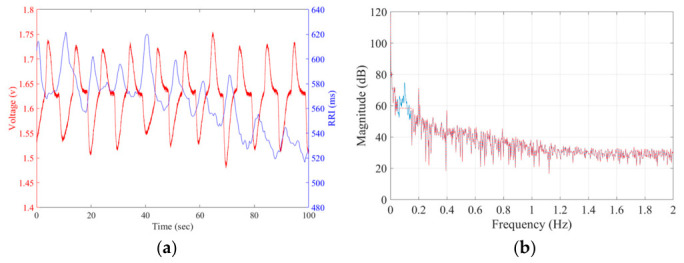
(**a**) The raw RRI signal (blue) coupling with the CBR signal (red); (**b**) the spectrum of the raw RRI signal (blue line) and the spectrum of the RRI signal upon removing the RSA energy (red dotted line).

Raw RRI or PPI sequences are typically represented as RRI(n) or PPI(n). The independent variable “n” of two sequences was transferred to a time variable “t”. Then, the raw RRI(t) and PPI(t) sequences were resampled to a uniform frequency of 4 Hz using a commonly used second-order polynomial interpolation method. This method fits a quadratic function to raw RRI(t) and PPI(t) signals, ensuring a smooth and accurate interpolation of values at the desired sampling rate. Figure 4a shows the raw RRI(t) (blue line) and respiratory signal (red line) within 10 s under a CBR of 6 BRPM. The left and right vertical axes represent the amplitude of the respiratory signal and the time of the RRI signal, respectively. The raw RRI(t) signal coupling with RSA energy shows the respiratory rhythms. Figure 4b shows the spectrum (PSD_Raw−RRI_(f), blue line) of the raw RRI(t) signal, which exhibits a spike at 0.1 Hz. The spectrum (PSD_Filtered−RRI_(f)_,_ red dot line) was obtained by replacing PSD_Raw–RRI(f)_ within the range of 0.1 Hz ± 0.05 Hz with the average of PSD_Raw–RRI_ (0.05) and PSD_Raw–RRI_ (0.15). Then, the inverse discrete-time Fourier transform was used to obtain the denoised RRI signals as the target RRI signal. For 18 BRPM, the spectrum (PSD_Filtered−RRI_(f)) was obtained by replacing PSD_Raw–RRI(f)_ within the range of 0.3 Hz ± 0.05 Hz with the average of PSD_Raw–RRI_ (0.25) and PSD_Raw–RRI_ (0.35). For 30 BRPM, the spectrum (PSD_Filtered−RRI_(f)) was obtained by replacing PSD_Raw–RRI(f)_ within the range of 0.5 Hz ± 0.05 Hz with the average of PSD_Raw–RRI_ (0.45) and PSD_Raw–RRI_ (0.55).

**Figure 5:**
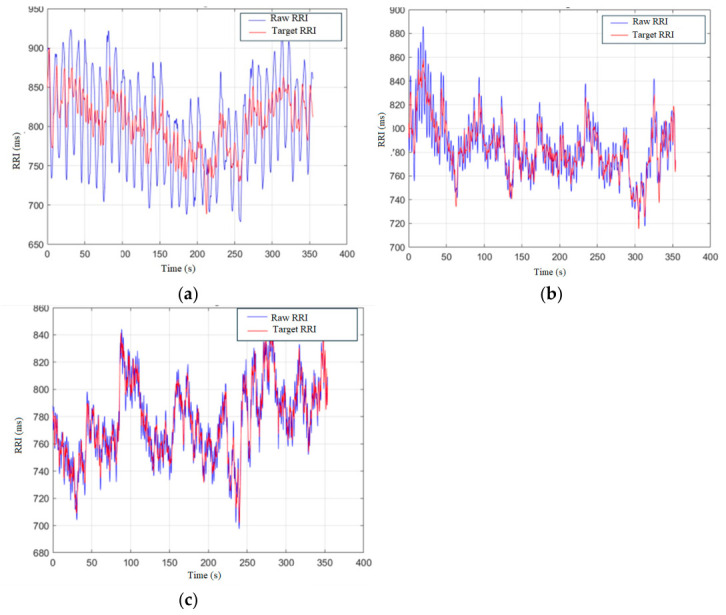
Raw RRI signals (blue line) and target RRI signals (red line) under different CBRs: (**a**) 6 BRPM, (**b**) 18 BRPM, and (**c**) 30 BRPM.

Figure 5 shows the raw and target RRI(t) signals under (a) 6 BRPM, (b) 18 BRPM, and (c) 30 BRPM, respectively. We can observe that the RSA energies of the RRI signals under 6 BRPM and 18 BRPM have significant attenuations. However, the raw and target RRI signals under 30 BRPM are very close.

### 2.5. Data Segmentation

In this study, both ECG and PPG signals were recorded continuously for 6 min under an SBR and the different CBRs of 6, 18, and 30 BRPM. To enhance the dataset robustness and support ARMA training, a segmentation strategy was applied. Specifically, each recording was divided into 1 min segments with forward–backward overlapping with a 30 s sliding window, preserving temporal continuity while increasing the sample count. Each measurement comprised 10 samples; each sample contained 240 data points for RRI(t) and PPI(t). Each subject contributed to 8 measurements, which are mentioned in Section 2.1 (Experimental Protocol). In such measurements, a subject yielded a total of 80 samples, and 1520 samples overall (10 samples × 8 measurements × 19 subjects). The segmentation approach thus significantly augmented the dataset without compromising the physiological signal integrity, enabling the improved modeling of the temporal dynamics between the PPI(t) and RRI(t) signals.

### 2.6. ARMA Model

We used an ARMA model to predict the PPI ($\tilde{PPI}(t)$) signal. Equation (4) is the ARMA model, *y*(t) the target RRI(t), and *u*(t) the raw PPI(t).

(4) $$y\left(t\right)+a_{1}y\left(t-1\right)+\cdots +a_{na}\;y\left(t-n_{a}\right)=b_{1}u\left(t-n_{k}\right)+\cdots +b_{nb}u\left(t-n_{k}-n_{b}+1\right),\;$$

where *n_a_* and *n_b_* are the orders of the autoregressive model and moving average model, and *n_k_* is the delay time of the input signal. In this study, *n_k_* is defined as 0, and *n_a_* and *n_b_* are the same. We designed the individual subject and general models to analyze the performance of ARMA time-series models. For each ARMA_individual_model_, the training samples were the maximum MAE_RAW_s, totaling 10 samples. The remaining 70 samples were used for testing. Table 1 and Table 2 present the MAE_RAW_s (in milliseconds) for subjects 1–10 and 11–19 under an SBR and different CBRs. For subjects 1–4, 6, 8, 11, 12, 14, 15, 17, and 18, the maximum MAE_RAW_s occurred at 6 BRPM during the first measurement. In contrast, for subjects 5, 7, 9, 10, 13, 16, and 19, the maximum MAE_RAW_s occurred at 6 BRPM during the second measurement.

To determine the optimal order for each ARMA_individual_model_, the model orders were scanned from 1 to 20. Table 3 and Table 4 present the mean absolute errors between the target RRI(t) and the predicted $\tilde{PPI}(t)$, denoted as MAE_Subject-Models_. For each ARMA_individual_model_ under different orders, if $\tilde{PPI}(t)$ did not converge, the corresponding MAE_Subject-Model_ is indicated by “--”. The order that yielded the minimum MAE_Subject-Model_ was selected as the optimal order of this ARMA_individual_model_ to predict the PPI(t) for that subject. Thus, there were 19 ARMA_individual_model_s.

For the ARMA_general_model_, a histogram method was used to compute the accumulated MAE_Subject-Model_s across different orders, as shown in Table 3 and Table 4. The order with the smallest accumulated MAE_Subject-Model_ was defined as the optimal order of the ARMA_general_model_. Subsequently, a total of 190 samples with the maximum MAE_RAW_s from the 19 subjects were used to train the ARMA_general_model_, while the remaining 1330 samples were used for testing.

## 3. Results

### 3.1. Individual Subject ARMA Models

In Table 1, for subjects 1–4, 6, 8, 11, 12, 14, 15, 17, and 18, the maximum MAE_RAW_s were obtained at 6 BRPM in the first measurement. In Table 2, for subjects 5, 7, 9, 10, 13, 16, and 19, the maximum MAE_RAW_s were found at 6 BRPM in the second measurement. Thus, these 10 samples of the subject at 6 BRPM were used to train their ARMA_individual_model_, and the remaining 70 samples of the subject were used to test the model. To determine the optimal order of each ARMA_individual_model_, we scanned orders from 1 to 20. Their MAE_Subject-Model_s under different orders are shown in Table 3 and Table 4. When the order had the minimum MAE_Subject-Model_, the model of this order would be used to predict the PPI(t). Subjects 1–19 used the 20th, 12th 19th, 16th, 19th, 15th, 3rd, 9th, 12th, 11th, 11th, 20th, 4th, 3rd, 15th, 15th, 3rd, 4th, and 19th order models, respectively. Table 5 and Table 6 show the MAE_Subject-Model_s of ARMA_individual_model_s for subjects 1 to 10, and subjects 11 to 19, respectively. For subjects 1 to 10, the MAE_Subject-Model_s are compared with MAE_RAW_s in Table 1, whose attenuations (ATTs) are from 9.5% to 42.3%. For subjects 11 to 19, except subject 18, the MAE_Subject-Model_s are compared with MAE_RAW_s in Table 2, whose ATTs are from 6.7% to 46.7%. The ATT is defined in Equation (5).

(5) $$ATT\;\%=\frac{{Parameter}_{Denoising\;before}-{Parameter}_{Denoising\;after}}{{Parameter}_{Denoising\;before}}\times 100.$$

**Figure 6:**
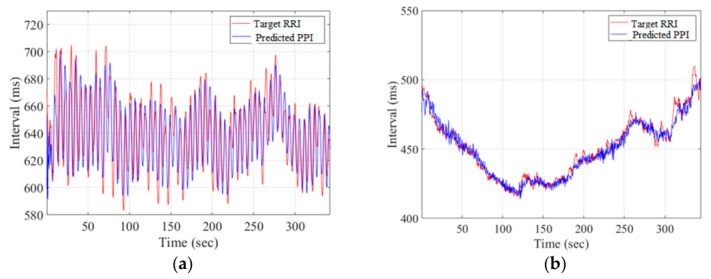
The target RRI(t) and $\tilde{PPI}(t)$ of subject 9: (**a**) an SBR; (**b**) the CBR of 30 BRPM.

Figure 6a shows the target RRI (red) and the predicted $\tilde{PPI}(t)$ (blue) of subject 9 under an SBR, where the MAE_Subject-Model_ is 12.65 ms. Figure 6b shows the target RRI (red) and the predicted $\tilde{PPI}(t)$ (blue) of subject 9 under a CBR of 30 BRPM, where the MAE_Subject-Model_ is 3.45 ms.

### 3.2. General ARMA Model

**Figure 7:**
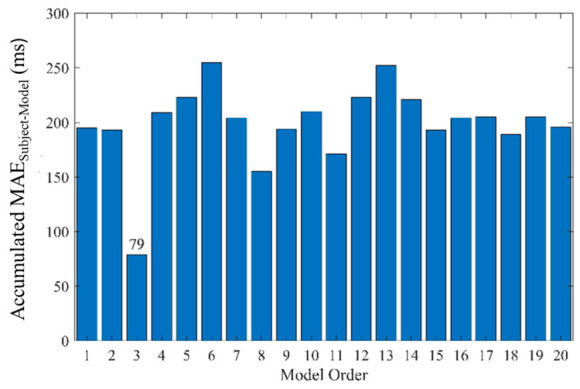
A histogram to illustrate the accumulated MAE_Subject-Model_s at different orders according to Table 3 and Table 4.

We used a histogram to illustrate the accumulated MAE_Subject-Model_s at different orders according to Table 3 and Table 4. The third order has the smallest MAE_Subject-Model_, 79 ms, as shown in Figure 7. Thus, the order of the general model is 3. The training samples were the measurements for each subject with the maximum MAE_RAW_s, and the number of training samples was 190. The other 1330 samples were used to test the ARMAgeneral_model. Table 7 and Table 8 show the MAEs between the target RRI(t) and the predicted $\tilde{PPI}(t)$ (MAE_General Model_s) for subjects 1 to 10 at SBR and the different CBRs, and for subjects 11 to 19. For subjects 1 to 10 except subject 2, the MAE_General Model_s are compared with MEA_RAW_s in Table 1, whose ATTs are from 11.8% to 39.2%. For subjects 11 to 19, except subject 18, the MAE_General Model_s are compared with MEA_RAW_s in Table 2, whose ATTs are from 2.3% to 47.1%.

### 3.3. ARMA_individual_model_s Compared with ARMA_general_model_

We proposed 19 individual subject models and a general model to remove the RSA energy coupled in the raw PPI signals. A t-test was used to compare the performance of individual subject models and the general model. Table 9 shows the summarized performance of the individual subject models and the general model. The means ± standard deviations of the MAE_Subject-Model_s and MAE_General-Model_ by the individual subject models and the general model for 19 subjects are 132.5 ± 59.1 ms and 137.8 ± 67.8 ms, respectively. Moreover, the means ± standard deviations of the ATT of the two models are 28.5% ± 13.1% and 27.8% ± 12.6%, respectively. The *p*-values of the MAEs and attenuations of the two models are 0.1827 and 0.6204, respectively. Thus, the performance of the two methods shows no significant difference.

In this study, CBRs were defined at 6 BRPM, 18 BRPM, and 30 BRPM, approximately 0.1 HZ, 0.3 Hz, and 0.5 Hz. The VLF, LF, and HF are the frequency-domain parameters of PRV, and their bandwidths are 0.003–0.04 Hz, 0.04–0.15 Hz, and 0.15–0.4 Hz, respectively. Thus, only the LF and HF parameters are affected by the designed CBRs of 6 BRPM and 18 BRPM. Table 10 shows that the mean absolute percentage errors (MAPEs) of the three frequency-domain parameters of PRV extracted from the raw PPI(t) and $\tilde{PPI}(t)$ predicted by ARMA_individual_model_s under eight measurements are represented by $\bar{VLF}$, $\bar{LF}$, and $\bar{HF}$, and $\tilde{VLF}$, $\tilde{LF}$, and $\tilde{HF}$. The MAPE is defined in Equation (5). Then, a t-test was used to compare the differences in the three frequency-domain parameters in two models. The results are shown in Table 10, where the MAPE of $\bar{VLF}$ is significantly smaller than that of $\tilde{VLF}$, the *p*-value being below 0.0001. This can be attributed to two issues. First, the VLF is not disturbed by RSA. In Table 1 and Table 2, the MAE_RAW_s at SBR are lower than 6.58 ms. Thus, the $\bar{VLF}$ extracted from the raw PPI(t) is very close to the truthful energy of the VLF extracted from the target RRI(t). Second, in Table 5 and Table 6, the maximum MAE_Subject-Model_s were obtained at SBR, and they were larger than 4.25 ms. Thus, the MAPEs of the $\bar{VLF}$ and $\tilde{VLF}$ are 1.53 ± 0.99% and 8.80 ± 10.59%, and the *p*-value between the MAPEs of the $\bar{VLF}$ and $\tilde{VLF}$ is lower than 0.0001. The $\tilde{VLF}$ is significantly worse than the $\bar{VLF}$. This result indicates that, when the proposed ARMA_individual_model_s removed RSA energy from the raw PPI signals, it may also have removed or distorted the true VLF energy. Moreover, the MAPE of the $\bar{LF}$ is significantly larger than that of the $\tilde{LF}$, with the *p*-value being below 0.0001. The reason is that the RSA of 6 BRPM interfered with the raw PPI(t), and $\tilde{PPI}(t)$ was removed from the respiratory signal by the ARMA_individual_model_s. In Table 11, the total MAE_RAW_s under RSA of 6 BRPM is significantly larger than MAE_Subject-Model_s, with a *p*-value of 0.000. This result shows that the proposed ARMA_individual_model_s could remove the RSA energy of raw PPI signals. ATT approaches 85.4% ± 15.1%. However, the MAPEs of $\bar{HF}$ and $\tilde{HF}$ are not significantly different, with a *p*-value of 0.5377. The reason is that HF parameters would be disturbed by RSA at 18 BRPM. In Table 11, the MAE_RAW_s under RSA at 18 BRPM are not significantly different from the MAE_Subject-Model_s, with a *p*-value of 0.2409. However, the ATT approached 16.5% ± 33.2%.

(6) $$MAPE=\frac{\;1\;}{\;8\;}\sum_{i=1}^{8}\frac{\left|Raw\;or\;Predict\;Parameter-Target\;Parameter\right|}{actual}*100.$$

## 4. Discussion

RSA is heart rate variability in synchrony with respiration, affecting the accuracy of HRV for evaluating the balance of ANS. The RRI(n) derived from an ECG is shortened and prolonged during inspiration and expiration, respectively [26]. Some studies have proposed methods for extracting respiratory rates (RRs) from RRI and R-wave amplitude signals [27,28]. Thus, when the PPI(t) or RRI(t) extracted from wearable devices is used for the long-term monitoring of PRV or HRV, RSA must be moved from raw PPI(t) or RRI(t) to increase the accuracy and stability of the parameters of PRV or HRV. However, because the raw PPI(t) or RRI(t) is modulated by RSA, it cannot be filtered using the traditional linear IIR or FIR filter. In this study, we used the spectrum identification method to remove RSA from the raw RRI(t). However, this method cannot be implemented in a wearable device for denoising RSA of PPI(t) because the real respiratory rate is unknown. Thus, we proposed 19 ARMA_individual_model_s and an ARMA_general_model_ for removing the RSA energy coupled in the raw PPI(t) signals for real-time PRV analysis.

The waveforms of PPG are easily affected by vascular compliance, peripheral vascular resistance, sensor displacement, skin tone, ambient light variations, and motion artifacts [11,29]. Peralta et al. evaluated the precision of PRV with HRV derived from ECG as the reference. PRV derived from PPI(n) was extracted from the five different points of the PPG waveform. It was found that the middle-amplitude point, apex point of the first differentiation, and tangent intersection point were the most suitable fiducial points for PRV analysis, resulting in the lowest relative errors between the PRV and HRV parameters and higher correlation coefficients and reliability indices [14]. Thus, we used the maximum-slope point of the PPG waveform to detect PPI(n).

PPI signals are very close to RRI signals in stationary subjects. As shown in Table 1 and Table 2, the MAE_RAW_ for subject 2 is 13.29 ms and that for subject 17 is 1.87 ms under an SBR. Thus, spontaneous respiratory signals could be extracted from RRI and/or R-wave amplitude (RWA) signals. However, the ANS regulates both heart rate (HR) and breathing rate (BR) [5]. Therefore, the VLF and LF bands also contain components related to BR. For HRV analysis, the BR-related energy is typically not removed from RRI signals. In contrast, under the CBRs, RSA energy may distort HRV analysis; therefore, it should be removed from the raw RRI signals prior to HRV analysis. This is because the bandwidths of SBR and HR are significantly different, allowing simpler approaches, such as high-pass filtering, to effectively remove SBR energy. However, when CBR overlaps (aliases) with HR, removing the coupled CBR energy from the RRI signal becomes challenging. According to previous studies, methods such as adaptive filtering [15,16], PCA [17], and EMD [18,19] require respiratory signals as reference inputs. In this study, we propose 19 ARMA_individual_model_s and an ARMA_general_model_ to remove RSA energy coupled in the PPI signal. This method does not require respiratory signal measurements and demonstrates generalization across subjects, making it more practical for real-world applications. Finally, because the CBRs were predefined in our experiment, a spectral method could be used to remove the corresponding RSA energy. However, in real-world applications, the actual CBRs are typically unknown. Therefore, spectral methods cannot be reliably used to remove unknown RSA energy.

Previous studies used adaptive filtering, PCA, and EMD to remove the energy of RSA from raw RRI signals. Table 12 shows the results, advantages, and limitations of these studies as the benchmark of our study. The same limitation of these previous studies [15,16,17,18,19] was that the synchronous respiratory signal had to be measured. Thus, these methods are difficult to apply in the real world. Cassani et al. [15] used an adaptive filter for removal. However, their method eliminated RSA energy from the raw RRI signal under a CBR of 15 BRPM (0.25 Hz), where the coupling occurs only within the HF band. The ATT of the HF band approached 40.3%, but the energy of the LF band increased from 12.7 ms^2^ to 47.8 ms^2^. Thus, this method would also remove or disturb the true energy of the LF band, like our proposed method. The energy of the VLF band increased from 1.53 ± 0.99 ms^2^ to 8.80 ± 10.59 ms^2^. However, under CBRs of 6 BRPM and 18 BRPM, the ATTs of the LF and HF bands approach 85.4% ± 15.1% and 16.5% ± 33.2%. Thus, our method performs better than the method of Cassani et al. [15]. Tiinanen et al. [16] also used adaptive filtering to remove RSA energy from RRI signals. However, the ATTs of the LF and HF bands only approached 15.4% and 6.9%, which are worse than those obtained with our method, which approached 85.4% ± 15.1% and 16.5% ± 33.2%. Balocchi et al. [18] used the EMD method to decompose the RRI signal and search the respiratory signal from the first intrinsic model function (IMF 1). This method required the post-selection of components, and needed the respiratory signal as a reference. The ATT of LF/HF only approached 3.7%. Tiinanen et al. [17] used PCA and adaptive filtering to remove SBR energy from the raw RRI signal. Both the LF and HF bands showed significant differences compared with the raw RRI signal. In our method, only the LF band extracted from the ARMA_individual_model_ is significantly different from that of the raw RRI signals. A major advantage of the proposed method is that it does not require synchronous respiratory measurements. Therefore, it can be more easily implemented in real-world applications.

A critical aspect of validating the performance of removing the RSA energy coupled in the raw PPI(t) using the individual subject models is to understand how timing errors propagate into the VLF, LF, and HF metrics of PRV in Table 10. Small perturbations of MAE_RAW_s can disproportionately affect frequency-domain parameters, particularly the HF parameter, which represents parasympathetic modulation. Our studies showed that, while the RSA energy under lower BRPM severely distorts PRV spectra, the ARMA_individual_model_s maintain spectral errors within physiologically acceptable limits, with most deviations being below 10–12%. The individual subject models significantly reduce the MAPEs of $\bar{LF}$ from 1066.19 ± 875.90 (%) to 88.45 ± 49.21 (%) of $\tilde{LF}$. In Table 9, the results indicate that the performance of the general model is close to that of the individual subject models. Its advantage is not only correcting individual intervals but also preserving the temporal variability structure required for reliable PRV spectral estimation. We used the maximum-slope point of the PPG waveform to define the pulse position as the PPI(n) for reducing onset-detection ambiguity. These mechanical and peripheral delays [30] cannot be fully eliminated, meaning that ARMA models can substantially reduce but not completely remove RSA interference, causing timing errors in $\tilde{PPI}(t)$.

In this study, ECG and PPG signals were measured in an ideal laboratory environment, where conventional methods can reliably extract the internal beat interval (IBI) from ECG and PPG signals. In real-world wearable settings, PPG signals contain motion artifacts, baseline drift, and pulse-shape distortions that make accurate IBI extraction more challenging—conditions that motivate the use of maximum likelihood estimation or least squares estimation. Thus, the limitations of this study are as follows. First, although the study incorporated three breathing rates (6, 18, and 30 BRPM), all recordings were still collected under controlled laboratory conditions in healthy adults. However, in real practice, users breathe irregularly, their postures change, and the PPG signal couples with substantial motion artifacts. These problems will reduce the performance for denoising RSA of raw PPI(t). Second, the models were validated only on internally collected data, and no external independent dataset was used, which limits generalizability to other populations, devices, and recording environments. Third, the study did not include participants with cardiovascular or autonomic dysfunction, who often exhibit altered pulse morphology and greater signal variability; performance in these groups remains unknown. Finally, although the models significantly denoised RSA obstruction from raw PPI(t), the $\tilde{PPI}(t)$ preserved PRV spectral content (VLF, LF, and HF) which remained sensitive to residual timing errors, indicating the need for further refinement. Future work will expand the dataset to include diverse populations, such as elderly subjects or patients with known cardiovascular conditions. Moreover, measurements should be taken in different contexts, such as ambulatory recordings, pathological cohorts, multi-center datasets, and diverse wearable sensor platforms, to establish broader applicability beyond controlled laboratory conditions.

## 5. Conclusions

This study used 19 ARMA_individual_model_s and the ARMA_general_model_ to remove the RSA energy coupled in raw PPI(t) signals. Their performance did not significantly differ. For the raw RRI(t) signals, the spectral method was used to remove the coupling RSA energy. The filtered RRI(t) signals were the target RRI(t). The results showed that MAE_Subject-Model_s and MAE_General-Model_ showed significant decreases compared with MAE_RAW_s. The MAPEs of $\tilde{LF}$ were also significantly lower than those of $\bar{LF}$. The MAPEs of $\tilde{HF}$ also decreased compared with those of $\bar{HF}$ but not significantly. Finally, the proposed ARMA_general_model_ can be implemented in wearable devices for PRV applications in the future.

## Figures and Tables

**Table 1:** MAE_RAW_s (ms) of subjects 1 to 10 under an SBR and different CBRs.

		Sub. 1	Sub. 2	Sub. 3	Sub. 4	Sub. 5	Sub. 6	Sub. 7	Sub. 8	Sub. 9	Sub. 10
First Meas.	SBR	2.68	13.29	5.23	5.54	5.99	2.95	2.41	2.49	4.55	3.84
	6 BRPM	45.72	66.78	82.37	49.19	60.79	18.89	69.06	35.46	10.11	58.11
	18 BRPM	13.69	33.41	24.35	13.39	37.24	11.23	12.69	9.34	8.37	20.98
	30 BRPM	8.031	22.06	11.425	15.39	29.68	7.50	8.10	8.49	2.47	13.04
Second Meas.	SBR	3.03	4.435	3.98	1.98	6.58	2.43	2.61	1.92	2.44	3.65
	6 BRPM	45.24	60.97	76.40	16.48	68.86	13.82	94.26	32.33	23.48	61.81
	18 BRPM	12.33	28.64	24.82	6.89	34.46	8.59	14.12	11.81	6.16	21.72
	30 BRPM	9.225	17.34	11.50	6.10	21.02	5.36	7.11	7.60	5.11	13.94
Total		139.95	246.93	240.07	114.97	264.62	70.80	210.37	109.45	62.70	197.09
Mean ±SD		17.5 ±16.6	30.8 ±20.8	30.0 ±29.4	14.3 ±14.0	33.0 ±21.3	8.9 ± 5.3	26.3 ± 32.8	13.7 ± 12.1	7.8 ± 6.4	24.6 ± 21.3

SD: standard deviation.

**Table 2:** MAE_RAW_s (ms) of subjects 11 to 19 under an SBR and different CBRs.

		Sub. 11	Sub. 12	Sub. 13	Sub. 14	Sub. 15	Sub. 16	Sub. 17	Sub. 18	Sub. 19
First Meas.	SBR	2.52	3.22	2.07	4.88	5.68	5.97	1.87	5.39	3.45
	6 BRPM	71.94	40.29	50.09	101.13	72.77	71.51	29.74	88.13	41.63
	18 BRPM	12.93	13.00	18.74	26.33	16.62	49.94	14.66	31.44	20.94
	30 BRPM	6.85	8.71	8.05	21.25	10.12	18.09	12.21	16.37	8.62
Second Meas.	SBR	2.82	2.61	4.26	5.17	3.58	4.06	4.06	4.84	5.65
	6 BRPM	34.75	35.29	96.28	65.55	69.91	81.21	17.05	46.41	80.50
	18 BRPM	8.06	11.16	16.14	28.10	17.32	25.33	11.79	28.18	25.73
	30 BRPM	10.49	7.80	7.64	17.38	11.84	20.11	5.88	21.61	11.17
Total		150.39	122.08	203.27	269.78	207.84	276.23	97.27	242.37	197.68
Mean ±SD		18.8 ± 22.24	15.3 ± 13.5	25.4 ± 30.4	33.7 ± 31.1	25.9 ± 26.6	34.5 ± 27.6	12.2 ± 8.29	30.3 ± 25.4	24.7 ± 24.2

SD: standard deviation.

**Table 3:** MAE_Subject-Model_s (ms) of subjects 1 to 10 under different orders. The orders with the minimum MAE_Subject-Model_s are the 20th order for subject 1, 12th order for subject 2, 19th order for subject 3, 16th order for subject 4, 19th order for subject 5, 15th order for subject 6, 3rd order for subject 7, 9th order for subject 8, 12th order for subject 9, and 11th order for subject 10.

Order of Model	Sub. 1	Sub. 2	Sub. 3	Sub. 4	Sub. 5	Sub. 6	Sub. 7	Sub. 8	Sub. 9	Sub. 10
1	100.1	54.34	60.64	34.07	61.19	36.38	93.70	35.57	37.38	54.57
2	--	39.60	109.70	54.45	95.39	--	61.66	--	--	41.58
3	8.69	32.83	25.20	23.84	18.39	8.07	17.33	10.21	7.03	15.48
4	14.41	40.60	31.57	--	32.10	---	24.51	20.76	87.01	--
5	--	46.50	--	--	17.10	--	--	--	8.03	15.99
6	--	45.91	--	28.58	12.68	--	--	--	8.49	29.81
7	--	33.95	34.60	27.05	11.37	-	-	9.49	6.37	25.98
8	--	28.38	30.84	--	11.25	--	--	--	--	18.64
9	9.22	30.90	--	26.68	--	--	--	8.65	--	23.54
10	8.52	28.93	--	27.55	12.71	--	--	9.64	--	--
11	7.52	26.62	--	27.77	10.53	9.33	--	--	--	15.07
12	--	25.10	--	29.05	--	--	--	10.52	5.81	--
13	--	30.62	23.24	--	10.39	--	--	--	--	--
14	--	35.68	26.11	25.25	--	8.32	17.76	--	--	--
15	10.27	34.08	--	23.10	--	6.35	--	--	--	--
16	23.82	30.69	--	21.42	--	6.76	17.83	--	--	--
17	7.76	44.53	--	21.69	--	--	18.43	--	--	--
18	11.07	39.27	--	21.49	--	8.71	18.37	--	--	--
19	--	40.10	19.43	911.49	10.33	198.35	20.21	--	--	--
20	7.48	149.18	534.83	22.03	10.71	--	18.86	12.40	--	--

“--” indicates that the ARMA model did not converge.

**Table 4:** MAE_Subject-Model_s (ms) of subjects 11 to 19 under different orders. The orders with the minimum MAE_Subject-Model_s are the 11th order for subject 11, 20th order for subject 12, 4th order for subject 13, 3rd order for subject 14, 15th order for subject 15, 15th order for subject 16, 3rd order for subject 17, 4th order for subject 18, and 9th order for subject 19.

Order of Model	Sub. 11	Sub. 12	Sub. 13	Sub. 14	Sub. 15	Sub. 16	Sub. 17	Sub. 18	Sub. 19
1	384.59	24.165	173.08	48.96	83.27	57.10	23.71	60.10	--
2	92.24	34.05	136.73	99.49	95.29	109.57	28.86	57.02	58.54
3	13.31	8.47	14.32	24.08	15.83	28.60	11.73	53.38	21.56
4	14.89	23.43	13.97	--	--	--	15.91	53.02	26.64
5	--	--	14.54	55.38	--	44.80	16.07	53.70	18.84
6	--	9.30	--	--	--	--	--	55.13	19.70
7	--	10.02	--	--	18.97	--	14.66	56.83	31.31
8	14.49	9.18	15.32	--	19.12	24.39	13.47	56.95	17.29
9	12.49	10.58	-	102.41	--	--	--	56.62	16.99
10	17.14	--	--	768.88	--	--	14.16	58.13	--
11	11.27	--	--	--	--	21.27	--	56.75	19.05
12	13.58	--	--	--	--	20.50	--	60.96	29.79
13	--	10.62	--	-	--	20.42	--	58.36	--
14	19.47	--	--	--	14.83	--	18.03	59.96	--
15	--	16.67	--	--	14.82	19.41	--	58.76	17.02
16	--	--	--	--	15.72	19.46	--	61.38	25.23
17	--	9.83	--	--	18.44	19.77	--	71.59	21.08
18	--	9.76	--	--	18.34	--	14.38	62.54	19.25
19	--	6.55	--	--	--	--	---	57.40	--
20	--	6.14	--	--	--	19.96	--	58.70	--

“--” indicates that the ARMA model did not converge.

**Table 5:** MAE_Subject-Model_s (ms) by ARMA_individual_model_s for subjects 1 to 10 under an SBR and different CBRs.

		Sub. 1	Sub. 2	Sub. 3	Sub. 4	Sub. 5	Sub. 6	Sub. 7	Sub. 8	Sub. 9	Sub. 10
First Meas.	SBR	7.39	43.37	13.40	14.10	13.70	8.05	15.30	19.99	12.65	6.19
	6 BRPM	7.48	25.29	19.44	21.42	12.16	6.34	23.68	8.58	5.06	14.64
	18 BRPM	14.55	26.83	24.69	13.09	32.24	11.18	19.22	9.53	7.87	19.46
	30 BRPM	9.92	26.50	17.24	19.04	25.19	10.31	18.72	9.31	3.46	14.65
Second Meas.	SBR	10.75	34.62	18.82	5.12	12.27	5.44	14.61	13.23	6.34	7.84
	6 BRPM	10.95	27.34	21.37	9.82	9.90	6.34	17.25	7.27	5.83	15.03
	18 BRPM	12.72	27.92	25.72	7.80	30.62	9.52	19.60	11.96	6.45	19.94
	30 BRPM	12.08	21.03	18.82	8.82	16.50	6.89	16.27	8.14	6.40	16.52
Total		85.83	232.90	159.51	99.22	152.58	64.07	144.64	88.01	54.06	114.28
ATT (%)		38.7	xx	33.6	13.7	42.3	9.5	31.2	19.6	13.8	42.0
Mean ±SD		10.7 ± 2.3	29.1 ± 6.4	19.9 ± 3.72	12.4 ± 5.3	19.1 ± 8.4	8.0 ± 1.9	18.1 ± 2.7	11.0 ± 3.8	6.8 ± 2.5	14.3 ± 4.6

SD: standard deviation. ATT: attenuation. xx represents no ATT.

**Table 6:** MAE_Subject-Model_s (ms) by ARMA_individual_model_s for subjects 11 to 19 under an SBR and different CBRs.

		Sub. 11	Sub. 12	Sub. 13	Sub. 14	Sub. 15	Sub. 16	Sub. 17	Sub. 18	Sub. 19
First Meas.	SBR	20.75	8.74	12.33	14.13	10.34	12.98	7.66	78.35	28.09
	6 BRPM	11.29	6.07	9.86	24.10	14.82	26.23	11.70	52.97	16.86
	18 BRPM	12.26	12.69	20.29	33.05	19.38	45.80	16.95	49.06	17.63
	30 BRPM	10.28	10.14	13.19	31.70	18.68	24.12	13.58	20.32	19.65
Second Meas.	SBR	17.68	7.96	8.09	19.00	8.79	14.04	4.25	27.57	34.32
	6 BRPM	10.17	6.34	13.83	22.38	12.27	19.41	7.81	27.87	16.84
	18 BRPM	8.35	10.60	18.47	33.01	18.56	26.00	12.65	29.20	25.64
	30 BRPM	12.79	9.20	12.11	26.67	14.88	25.40	7.16	22.78	25.27
Total		103.57	71.75	108.18	204.03	117.73	193.99	81.76	309.18	184.32
ATT (%)		31.1	41.2	46.8	24.4	43.4	29.8	15.9	xx	6.8
Mean ±SD		12.9 ± 3.9	9.0 ± 2.1	13.5 ± 3.8	25.5 ± 6.5	14.7 ± 3.7	24.3 ± 9.5	10.2 ± 3.9	38.6 ± 18.7	23.0 ± 5.9

SD: standard deviation. ATT: attenuation. xx represents no ATT.

**Table 7:** MAE_General _Model_ (ms) for subjects 1 to 10 under an SBR and different CBRs.

		Sub. 1	Sub. 2	Sub. 3	Sub. 4	Sub. 5	Sub. 6	Sub. 7	Sub. 8	Sub. 9	Sub. 10
First Meas.	SBR	6.63	49.24	8.82	16.49	32.95	6.16	15.30	17.62	12.39	6.53
	6 BRPM	8.68	32.71	25.28	23.84	19.99	8.09	23.68	10.16	4.46	13.88
	18 BRPM	14.07	33.65	25.26	13.33	25.28	10.22	19.22	9.29	8.09	22.21
	30 BRPM	9.83	33.80	12.63	17.33	29.10	8.89	18.72	9.40	3.00	14.19
Second Meas.	SBR	9.59	42.60	12.37	5.04	28.20	4.05	14.60	11.72	5.66	9.11
	6 BRPM	12.15	34.32	21.59	10.10	18.29	7.21	17.25	8.53	7.04	15.48
	18 BRPM	12.63	34.50	26.21	7.52	24.92	8.44	19.60	11.70	6.13	22.81
	30 BRPM	11.54	26.34	14.54	7.70	19.14	5.97	16.27	8.25	6.00	16.30
Total		85.13	287.18	146.68	101.35	197.87	59.03	144.64	86.66	52.77	120.42
ATT (%)		39.2	xx	38.9	11.8	25.2	16.6	31.2	20.8	15.8	38.9
Mean ±SD		10.6 ± 2.3	35.9 ± 6.5	18.3 ± 6.5	12.7 ± 5.9	24.7 ± 4.9	7.4 ± 1.8	18.1 ± 2.7	10.8 ± 2.8	6.6 ± 2.6	15.1 ± 5.3

SD: standard deviation. ATT: attenuation. xx represents no ATT.

**Table 8:** MAE_General_Model_ (ms) for subjects 11 to 19 under an SBR and different CBRs.

		Sub. 11	Sub. 12	Sub. 13	Sub. 14	Sub. 15	Sub. 16	Sub. 17	Sub. 18	Sub. 19
First Meas.	SBR	15.76	8.74	12.13	14.13	9.65	11.14	7.66	78.35	31.35
	6 BRPM	13.36	8.51	10.13	24.10	15.83	31.14	11.70	53.35	16.70
	18 BRPM	12.12	13.08	20.13	33.05	19.56	49.25	16.95	49.10	16.11
	30 BRPM	9.47	9.89	12.94	31.70	17.79	20.98	13.58	20.57	18.18
Second Meas.	SBR	13.56	7.71	7.74	19.00	8.04	10.79	4.25	27.70	41.12
	6 BRPM	12.53	7.77	14.21	22.38	13.51	28.60	7.81	27.96	21.19
	18 BRPM	7.68	10.90	18.38	33.01	18.82	26.53	12.65	29.28	24.74
	30 BRPM	12.26	8.74	11.76	26.67	14.48	22.24	7.16	22.87	23.66
Total		96.75	75.3	107.43	204.03	117.67	200.67	81.76	309.18	193.06
ATT (%)		35.7	38.3	47.1	24.4	43.4	27.4	15.9	xx	2.3
Mean ±SD		12.1 ± 2.3	9.4 ± 1.7	13.4 ± 3.8	25.5 ± 6.5	14.7 ± 3.9	25.1 ± 11.5	10.2 ± 3.9	38.6 ± 18.7	24.1 ± 7.9

SD: standard deviation. ATT: attenuation. xx represents no ATT.

**Table 9:** The performance of ARMA_individual_model_s and ARMA_general_model_ for 19 subjects.

	ARMA_individual_model_s (Baseline)		ARMA_general_model_	
	MAE_Subject-Model_s (ms)	ATT (%)	MAE_General-Model_ (ms)	ATT (%)
Sub. 1	85.83	38.7	85.13	39.2
Sub. 2	232.90	xx	287.18	xx
Sub. 3	159.51	33.6	146.68	38.9
Sub. 4	99.22	13.7	101.35	11.8
Sub. 5	152.58	42.3	197.87	25.2
Sub. 6	64.07	9.5	59.03	16.6
Sub. 7	144.64	31.2	144.64	31.2
Sub. 8	88.01	19.6	86.66	20.8
Sub. 9	54.06	13.8	52.77	15.8
Sub. 10	114.28	42.0	120.42	38.9
Sub. 11	103.57	31.1	96.75	35.7
Sub. 12	71.75	41.2	75.3	38.3
Sub. 13	108.18	46.8	107.43	47.1
Sub. 14	204.03	24.4	204.03	24.4
Sub. 15	117.73	43.4	117.67	43.4
Sub. 16	193.99	29.8	200.67	27.4
Sub. 17	81.76	15.9	81.76	15.9
Sub. 18	257.54	xx	259.19	xx
Sub. 19	184.32	6.8	193.06	2.3
Mean ± SD	132.5 ± 59.1	28. 5 ± 13.1	137.8 ± 67.8	27.8 ± 12.6
*p*-value	0.182703314	0.620455165		

SD: standard deviation. ATT: attenuation. xx represents no ATT.

**Table 10:** MAPEs of $\bar{VLF}$, $\bar{LF}$, and $\bar{HF}$ extracted from the raw PPI signals, and $\tilde{VLF}$, $\tilde{LF}$, and $\tilde{HF}$ extracted from the predicted $\tilde{PPI}\left(t\right)$ signals by ARMA_individual_model_s of subjects 1 to 19.

Subject	$\mathrm{MAPEs}\;\mathrm{of}\;\bar{VLF}$(%) (Baseline)	$\mathrm{MAPEs}\;\mathrm{of}\;\tilde{VLF}$(%)	$\mathrm{MAPEs}\;\mathrm{of}\;\bar{LF}$(%) (Baseline)	$\mathrm{MAPEs}\;\mathrm{of}\;\tilde{LF}$(%)	$\mathrm{MAPEs}\;\mathrm{of}\;\bar{HF}$(%) (Baseline)	$\mathrm{MAPEs}\;\mathrm{of}\;\tilde{HF}$(%)
1	1.068	1.536	920.666	36.115	106.000	91.824
2	2.040	44.498	833.227	50.425	237.074	70.548
3	0.738	7.142	1515.260	141.083	123.867	91.647
4	0.538	1.374	654.108	163.986	144.087	112.497
5	0.915	3.550	1199.021	57.546	275.117	200.126
6	1.056	8.114	81.836	50.469	175.102	123.424
7	1.511	3.692	679.553	65.816	81.424	94.821
8	0.609	5.782	976.206	71.691	322.287	274.138
9	1.648	18.058	299.110	71.048	248.459	88.086
10	1.873	2.075	1086.752	68.856	203.209	152.156
11	3.323	10.116	395.484	42.898	284.672	197.311
12	1.165	4.586	1092.355	57.736	231.961	182.100
13	0.713	2.346	2420.390	85.396	132.993	134.229
14	2.851	3.557	2594.116	224.310	117.494	158.694
15	0.864	3.662	3570.882	148.342	226.550	194.095
16	1.338	1.944	554.215	45.179	167.506	135.355
17	1.516	2.066	729.854	137.573	219.869	225.023
18	0.914	24.619	464.181	80.395	57.087	97.438
19	4.429	18.529	190.536	81.590	164.982	65.364
Mean ± SD	1.53 ± 0.99	8.80 ± 10.59	1066.19 ± 875.90	88.45 ± 49.21	185.25 ± 71.06	141.52 ± 56.18
*ATT* (*%*)	xx		85.4% ± 15.1%		16.5% ± 33.2%	
*p*-value	0.000951375		0.000145577		0.537672806	

SD: standard deviation. ATT: attenuation. xx represents no ATT.

**Table 11:** MAE_RAW_s (ms) and MAE_Subject-Model_s (ms) under SBR and different CBRs.

	MAE_RAW_s (ms) (Baseline)				MAE_Subject-Model_s (ms)			
Breath Rate	SBR	6 BRPM	18 BRPM	30 BRPM	SBR	6 BRPM	18 BRPM	30 BRPM
Total	154.13	2084.31	730.64	454.68	618.25	586.01	760.50	603.73
Mean ± SD	4.1 ± 2.0	54.9 ± 24.9	19.2 ± 9.9	12.06 ± 6.3	16.3 ± 13.6	15.4 ± 9.3	20.0 ± 10.1	15.9 ± 6.9
*p*-value	0.000	0.000	0.2409	0.000				

SD: standard deviation.

**Table 12:** The proposed method compared with previous studies.

Ref.	Method	Source	Sub.	Data of 1 min	Advantages	Limitations
[18]	EMD	ECG and breathing signal	13	ATT of average frequency ratio is 3.7%	Works for nonlinear signals	Requires post-selection of components
[19]	EMD and frequency peak found in the breathing and PPI signals reflect RR and HR	PPG and Capnogram	42	RMSE of BR is 3.5 BRPM	Works for nonlinear signals	Requires post-selection of components
[17]	PCA	ECG and breathing signal	23	Group 1 (SBR < 0.15 Hz): LF and HF have significant differences from baseline Group 2 (SBR > 0.15 Hz): LF and HF have significant differences from baseline	Dynamically tracks respiration	Needs respiratory measurement
[15]	Adaptive filtering	ECG and breathing signal	1	CBR = 15 BRPM; LF increased from 12.7 ms^2^ to 47.8 ms^2^; HF decreased from 87.3 ms^2^ to 52.1 ms^2^; ATT of 40.3% CBR = 36.96 BRPM; LF changed from 79.3 to 79.1 ms^2^; HF changed from 20.6 ms^2^ to 20.9 ms^2^	Dynamically tracks respiration	Needs respiratory measurement
[16]	Adaptive filtering	ECG and breathing signal	24	ATTs of LF and HF bands are 15.4% and 6.9%	Dynamically tracks respiration	Needs respiratory measurement
Proposed method	ARMA_individual_models_	PPG	19	MAPEs of $\bar{LF}$ and $\tilde{LF}$ have significant differences; ATT is 85.4% ± 15.1%.MAPEs of $\bar{HF}$ and $\tilde{HF}$ do not have significant differences; ATT is 16.5% ± 33.2%.	1. Exhibits superior generalization performance under both SBR and CBRs of 6, 18, and 30 BRPM 2. Eliminates the need for synchronized respiratory measurements	1. Lower sensitivity for denoising RSA of 18 BRPM 2. When the ARMA_individual_model_s removed RSA energy from raw PPI signals, they may also have removed or distorted the true VLF energy

## Data Availability

Data are contained within the article.

## Abbreviations

The following abbreviations are used in this manuscript:

PPI	Pulse-to-Pulse Interval
RRI	R-wave to R-wave Interval
PRV	Pulse Rate Variability
HRV	Heart Rate Variability
ARMA	Autoregressive Moving Average
MAE	Mean Absolute Error
BRPM	Breathing Rate Per Minute
